# Femoral µCT Analysis, Mechanical Testing and Immunolocalization of Bone Proteins in β-Hydroxy β-Methylbutyrate (HMB) Supplemented Spiny Mouse in a Model of Pregnancy and Lactation-Associated Osteoporosis

**DOI:** 10.3390/jcm10214808

**Published:** 2021-10-20

**Authors:** Ewa Tomaszewska, Siemowit Muszyński, Janine Donaldson, Piotr Dobrowolski, Deepesh K. P. Chand, Agnieszka Tomczyk-Warunek, Monika Hułas-Stasiak, Iwona Puzio, Krzysztof Lamorski, Cezary Sławiński, Mirosław Jabłoński, Tomasz Blicharski

**Affiliations:** 1 Department of Animal Physiology, Faculty of Veterinary Medicine, University of Life Sciences in Lublin, Akademicka St. 12, 20-950 Lublin, Poland; deepesh.parsan@gmail.com (D.K.P.C.); iwona.puzio@up.lublin.pl (I.P.); 2 Department of Biophysics, Faculty of Environmental Biology, University of Life Sciences in Lublin, Akademicka St. 13, 20-950 Lublin, Poland; 3 School of Physiology, Faculty of Health Sciences, University of the Witwatersrand, 7 York Road, Parktown, Johannesburg 2193, South Africa; janine.donaldson@wits.ac.za; 4 Department of Functional Anatomy and Cytobiology, Faculty of Biology and Biotechnology, Maria Curie-Sklodowska University, Akademicka St. 19, 20-033 Lublin, Poland; piotr.dobrowolski@umcs.lublin.pl (P.D.); monhul@o2.pl (M.H.-S.); 5 Bohdan Dobrzański Institute of Agrophysics of the Polish Academy of Sciences, Doświadczalna St. 4, 20-290 Lublin, Poland; a.tomczykwarunek@gmail.com (A.T.-W.); k.lamorski@ipan.lublin.pl (K.L.); c.slawinski@ipan.lublin.pl (C.S.); 6 Chair and Department of Rehabilitation and Orthopaedics, Medical University in Lublin, Jaczewskiego St. 8, 20-090 Lublin, Poland; mbjablonski@gmail.com (M.J.); blicharski@vp.pl (T.B.)

**Keywords:** β-hydroxy β-methylbutyrate, pregnancy, lactation, bone, bone quality, bone proteins

## Abstract

A metabolite of leucine, ß-hydroxy-ß-methylbutyrate (HMB), used as a dietary supplement effects muscle tissue gain and bone tissue quality. Since there are no studies on the effects of HMB during pregnancy yet, the aim of the current study was to determine the effects of HMB supplementation during pregnancy on osteoporotic bone quality postpartum and post-lactation using spiny mice (*Acomys cahirinus*) as the animal models. The six-month-old dams were divided into four groups: pregnant and lactating controls, and pregnant and lactating HMB-treated (during the second trimester of pregnancy) females. The intensity of the immunoreaction of osteocalcin (OC), osteoprotegerin (OPG), bone morphogenetic protein 2 (BMP-2), tissue inhibitor of metalloproteinases 2 (TIMP-2), matrix metalloproteinase 8 and 13 (MMP-8 and MMP-13) and proteins involved in bone turnover, was measured in femoral trabecular and compact bone, as well as in the hyaline and epiphyseal cartilage of the femora. The analysis of the trabecular bone microarchitecture showed that the administration of HMB to pregnant females, by influencing the proteins responsible for bone cell activity and collagen remodeling, can provide protection from bone loss. Based on the results of the current study it can be assumed that HMB administration to pregnant females has a more positive impact on trabecular than compact bone.

## 1. Introduction

Osteoporosis is the most common age-related metabolic bone disease, which damages the microarchitecture of bone tissue and reduces bone strength enhancing the risk of fractures, including low-energy fractures. Osteoporosis also occurs at a young age, although to a lesser extent than in older persons. Pregnancy also influences the quality of bone tissue in women, thus, contributing to the occurrence of osteoporosis [1,2,3]. During pregnancy, especially during the third trimester, mammalian fetuses undergo intensive development, thus, there is a high demand for calcium. Beginning in the first trimester, pregnant women accumulate calcium throughout pregnancy. The calcium supply from the mother to the fetus is dependent on its resorption of the maternal skeleton, increased intestinal reabsorption and reduced urinary excretion [4,5,6]. Although these events are a physiological state, they enhance bone turnover in pregnant and lactating women and are linked with asymptomatic decrease in bone mineral density (BMD) in the mother. Recovery normally occurs after six months of lactation, but sometimes it can lead to pregnancy- and lactation-associated osteoporosis (PLO), a rare form of osteoporosis that is severe and has an ambiguous cause [7]. Young women with PLO have low-trauma or spontaneous fractures, most commonly multiple vertebral fractures, which occur during late pregnancy or lactation [8] as a result of a decrease in BMD and mechanical stresses during pregnancy [7]. Severe back pain, that negatively affects basic life functions, can manifest in the third trimester of pregnancy or in the few first months after delivery and is linked with a reduction in height following worsening thoracic kyphosis. PLO commonly appears for the first time during the first pregnancy and sporadically in subsequent pregnancies. Medical reports suggest that PLO usually occurs in women who suffer from pre-existing osteopenia [7,8,9,10,11].

Over the past few decades, the effects of ß-hydroxy-ß-methylbutyrate (HMB), an endogenous metabolite of the essential amino acid leucine, have been studied intensively in human and animal models. The half-life of HMB in the blood is comparable to that of leucine. HMB in muscle and liver cells is used in the synthesis of cholesterol [12]. Its anabolic and anti-catabolic properties show a protective effect against muscle damage [12,13]. Its regenerative properties have also been observed in the wound-healing processes including that of bone fractures [14,15,16]. However, all these studies present the effect of postnatal HMB use and the studies focused on HMB supplementation during pregnancy are still lacking. Existing data from animal models, especially pigs, show that HMB supplementation during pregnancy results in prenatal programming of the offspring. Prenatal HMB treatment has been shown to have a positive effect on the term body weight, muscle tissue [17] and bone tissue of offspring [18,19,20,21]; however HMB is still not recommended for pregnant women [22,23].

The pregnant spiny mouse (*Acomys cahirinus*) has been used as an animal model in numerous studies such as dielectric rhythm, a diet-induced type 2 diabetes, late-pregnancy development, parental behavior and programming. The spiny mouse is also considered as a suitable rodent model due to its long gestation period (38–39 days) [24,25,26]. A previous study involving HMB supplementation in pregnant spiny mice, showed that the administration of HMB significantly improved skeletal metabolic processes [27]. The prevalence of PLO during pregnancy highlights the need for possible effective methods of prevention and treatment of skeletal system disorders. Functional food or supplements affecting skeletal tissue remodeling and homeostasis can be useful for prevention before PLO. Based on previous results, HMB supplementation during pregnancy has been shown to have a positive effect, not only on the offspring, but also on the pregnant dam’s skeletal system [21,27,28,29]. Taking into account the influence of pregnancy on bone quality and the effect of HMB in protecting pregnant women against excessive bone loss, it seems reasonable to investigate the effects of HMB supplementation during pregnancy on the immunolocalization of selected proteins involved in bone metabolism in pregnancy and lactation. In general, bone processes involve bone formation and resorption, which are regulated by the receptor activator of nuclear factor kappa-Β ligand (RANKL)/osteoprotegerin (OPG) ratio expressed by osteoblast and osteoclasts; the modulation calcium metabolism regulated by osteocalcin expressed by osteoblasts; cartilage formation induced by bone morphogenetic protein (BMP) also responsible for bone formation; and extracellular matrix degradation by collagenases (matrix metalloproteinases, MMP) [30,31,32].

Therefore, the objective of this study was to examine the effect of HMB supplementation in pregnant dams on the intensity of immunoreaction and immunolocalization of osteoprotegerin (OPG), bone morphogenetic protein 2 (BMP-2), osteocalcin (OC), tissue inhibitor of metalloproteinases 2 (TIMP-2) and matrix metalloproteinase 8 and 13 (MMP-8, MMP-13) in the bone tissue of a pregnant and lactating animal model. In doing so, the present study examined (i) the changes in the intensity of immunoreaction and immunolocalization of the above-mentioned proteins in bone tissue using standard immunohistochemistry methods, (ii) the effect of HMB supplementation during the second trimester of pregnancy in the spiny mice on general bone quality, microarchitecture of trabecular bone and bone biomechanical properties. Together, these measurements should provide important information regarding outcomes of HMB supplementation during pregnancy, in relation to signals involved in general bone turnover of the pregnant dams.

## 2. Materials and Methods

The study was performed in accordance with EU Directive 2010/63/EU and approved by the II Local Ethical Committee in Lublin, Poland (# 55/2013 and 8/2014).

### 2.1. Animals and Experimental Design

Clinically healthy, mature (approx. 6 months of age at parturition) spiny mice (*Acomys cahirinus*) dams in their second pregnancy were used in this study (*n* = 32). The females were mated naturally at a ratio of 1:1 with healthy males. Pregnant spiny mice were randomly divided into four groups (*n* = 8 each): pregnant females which were euthanized just after delivery (the CONT-D group; as delivered dams) or at weaning (when pups were 21 days old), after the lactation period following delivery (the CONT-L group; as lactating dams); the other two groups of pregnant females supplemented between the 13th and 26th days of pregnancy with HMB at a dose of 0.02 g/kg b.w., were divided similarly, into females euthanized just after delivery (the HMB-D group; as delivered dams) while the others were euthanized at weaning (the HMB-L group; as lactating dams).

All delivered or lactating dams were kept in standardized cages individually (Velaz, Praha, Czech Republic) under the same laboratory conditions: a temperature of 22 ± 2 °C, humidity of 55–60%, 12 h:12 h light cycle, an airflow speed of 0.3 m/s, and free access to water. Dams were fed a standard pelleted laboratory rodents diet specified in the AIN-93M directive [33] twice a day, and all the dams were weighed daily. The daily amount of food consumed by the dams (15 g) consisted of three pellets: one given in the morning and two at noon. The HMB (0.02 g/kg b.w.; Lonza, Basel, Switzerland, purity ≥99%) was supplemented in one morning pellet between the 13th and 26th day of pregnancy to females from the HMB groups. The dose of HMB used was based on the literature data and previous studies from our own laboratory [19,34,35,36,37,38,39,40]. Females from all experimental groups were euthanized by CO_2_ inhalation. After euthanasia, both femora were dissected from spiny mice and remaining soft tissues were removed using a scalpel blade. The left femora were immediately put on ice and within two hours were subjected to mechanical testing, while the right femora after weighing and measuring for length were stored in 70% EtOH until µCT imaging.

### 2.2. MECHANICAL Testing

The three-point bending test was performed using a universal testing machine (ZwickRoell 005, ZwickRoell GmbH & Co., Ulm, Germany) with the loading rate of 2 mm/min. Recorded load–deformation curves and determined bone mid-diaphysis geometry parameters were used for the calculation of the following mechanical parameters: yield strength (determining the strength under elastic deformation), the ultimate fracture strength (the force recorded at the bone fracture), stiffness (describing the elastic behavior of the bone), Young’s modulus (describing the bending resistance of the bone), and work to fracture (equal to total energy absorbed by the bone during bending) [41].

### 2.3. Immunohistochemical Staining

Immediately after mechanical testing from femora stored on ice, the distal epiphysis was cut. Samples were fixed in phosphate-buffered paraformaldehyde (4% *v/v*, pH 7.0), then ethylene diamine tetra acetic acid (10% *w/v*, pH 7.4) solution was used for decalcification. Next, after the dehydration in ascending EtOH solutions (30–95%, *v/v*), samples were fixed with nonpolar solvents (Ottix Plus and Ottix Sharper, DiaPath, Martinengo, Italy), embedded in paraffin and cut using a microtome (HM360, Microm, Walldorf, Germany) into 5-μm thick sections. For immunohistochemistry, the following antibodies were diluted in Diamond antibody diluent (Cell Marque Corp., Rocklin, CA, USA) and were used as primary antibodies: rabbit polyclonal to osteoprotegerin (OPG; ab73400, Abcamab, Cambridge, UK, dilution 1:100); mouse monoclonal to osteocalcin (OC, orb259644, Biorbyt, Cambridge, UK, dilution 1:100); mouse monoclonal to tissue inhibitor of metalloproteinases 2 (TIMP-2; ab1828, Abcam, Cambridge, UK, dilution 1:100); rabbit monoclonal to bone morphogenetic protein 2 (BMP-2; pa5-85956 Invitrogen, Thermo Fisher, dilution 1:200); rabbit monoclonal to matrix metalloproteinase 8 (MMP-8; ab81286, Abcam, Cambridge, UK, dilution 1:200); rabbit polyclonal to matrix metalloproteinase 13 (MMP-13; ab75606, Abcam, Cambridge, UK, dilution 1:200). As a secondary antibody, a Bright Vision +Poly-HRP-Anti Ms/Rb IgG Biotin-free (Immunologic, Duiven, Netherlands) kit was used, while 3,3′-diaminobenzidine tetrahydrochloride (DAB, D5905, Sigma-Aldrich, St. Louis, MO, USA) was used as a substrate-staining chromogen. Counterstaining was performed with Mayer’s hematoxylin (MHS32-1L, Sigma-Aldrich) [42,43]. Negative control sections for each antibody were conducted by identical immunohistochemical staining excluding the primary antibody (Supplementary Data, Figure S1). The intensity of immunoreaction was measured using a CX43 microscope (Olympus, Tokyo, Japan), both by determining the percentage of cells with a positive response, and by the quantitative comparison of mean pixel intensity in the photomicrographs, which were firstly converted into negatives and then into 8-bit grey-scale digital images, with a scale from 0 (white pixel) to 255 (black pixel), where the higher the pixel value, the higher the intensity of the immunohistochemical reaction [29,44]. For the immunoreactive cell count procedure, four randomly selected areas of compact bone, trabecular bone and articular cartilage, respectively, were measured for each microscopic slide. The intensity of immunoreaction was measured in six randomly selected areas of the growth plate cartilage, and twelve randomly selected areas of the positive signal in compact bone, trabecular bone and articular cartilage. For compact and trabecular bone, the intensity of immunoreaction was measured separately for osteocytes and the bone matrix. All the analyses were carried out blindly using ImageJ software [45].

### 2.4. Micro Computed Tomography Analysis

To prevent bones from drying during X-ray computed tomography, scan bones were placed in sealed tubes maintaining a saturated EtOH vapor. A Nanotom 180S apparatus (GE Sensing & Inspection Technologies GmbH, Wunstorf, Germany) was used for scanning using the following settings: rotation step: X-ray source current: 250 µA; X-ray source voltage: 140 kV; rotation step: 0.24°; scan resolution: 11 µm. Grey-level 16-bit 3D images were reconstructed using DatosX 2.0 software (GE Sensing & Inspection Technologies GmbH, Wunstorf, Germany). For scan visualization and analysis, the following software was used: VG Studio Max (v. 2.0, Volume Graphics GmbH, Heidelberg, Germany), ImageJ (v. 1.53j, U.S. National Institutes of Health, Bethesda, MA, USA) and Avizo (v. 9, FEI, Hillsboro, Oregon, USA) [27]. The following regions of interest (ROI) were analyzed using BoneJ plugin for ImageJ software [46]: (i) transverse section of bone mid-diaphysis (measurements of bone cross-section diameters, cross-sectional area, cortical index and mean relative wall-thickness), and (ii) sagittal section of the middle part of lateral condyle (measurements of trabecular bone volume fraction, trabecular thickness and trabecular separation) [47].

### 2.5. Statistical Analysis

Data were analyzed using the GLM MIXED procedure of SAS (SAS Institute. Inc., Cary, NC, USA) with the supplementation (control, HMB) and period (delivery, lactation) and their interaction as fixed effects and spiny mouse as random effects. Means were compared using a two-way ANOVA; significant differences were separated using a Tukey’s post-hoc test. A *p*-value of <0.05 was considered statistically significant.

## 3. Results

### 3.1. Bone Analysis

**Figure 1:**
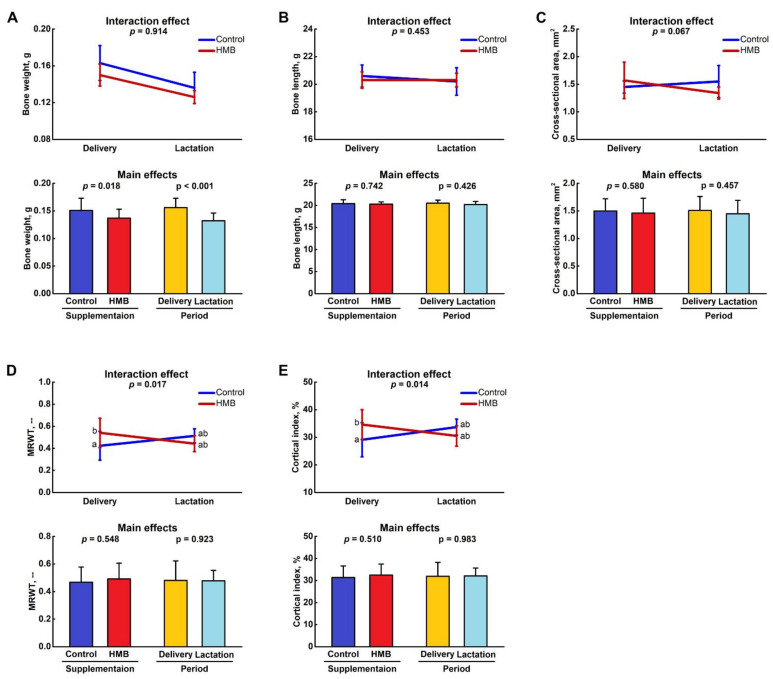
Effect of HMB supplementation (0.02 g/kg b.w.) during the middle trimester of pregnancy (13–26 d) on (**A**) weight, (**B**) length of femora as well as bone mid-diaphysis (**C**) cross-sectional area, (**D**) mean relative wall thickness (MRWT) and (**E**) cortical index of femora of spiny mice at delivery or after the lactation period. Figure shows LSMeans ± standard deviation as well as *p*-values of a two-way ANOVA with the supplementation (control, HMB) and period (delivery, lactation) and their interaction as fixed effects. Means with different letters in interaction effect plots differ significantly (Tukey’s post-hoc test).

**Figure 2:**
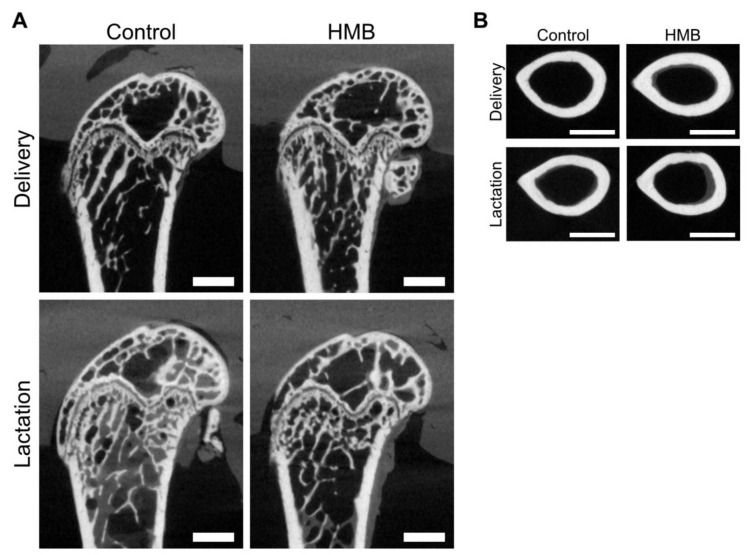
Representative µCT images of sagittal sections of the femur distal diaphysis (**A**) and transverse sections of the femur mid-diaphysis (**B**) from pregnant female controls (not receiving HMB) at delivery (CONT-D) or after the lactation period (CONT-L) and from pregnant HMB females (receiving HMB at a dose of 0.02 g/kg b.w. during the middle trimester of pregnancy) at delivery (HMB-D) or after the lactation period (HMB-L). Scale bars represent 1000 µm.

Figure 1 shows the weight, length and mid-diaphysis geometric parameters of the femora from the control and HMB-supplemented females. Femora weight was dependent both on the supplementation and period examined (*p* = 0.018 and *p* < 0.001, respectively; Figure 1A), while there was no effect on bone length (Figure 1B) or the bone mid-diaphysis cross-sectional area (Figure 1C). On the other hand, there was a significant interaction of supplementation and period on the mean relative wall thickness and cortical index, and there was a difference between HMB-treated and control dams on delivery (*p* = 0.017; Figure 1D and *p* = 0.014; Figure 1E, respectively). Figure 2B shows representative µCT images of cross-sections of the femoral mid-diaphysis in examined groups.

**Figure 3:**
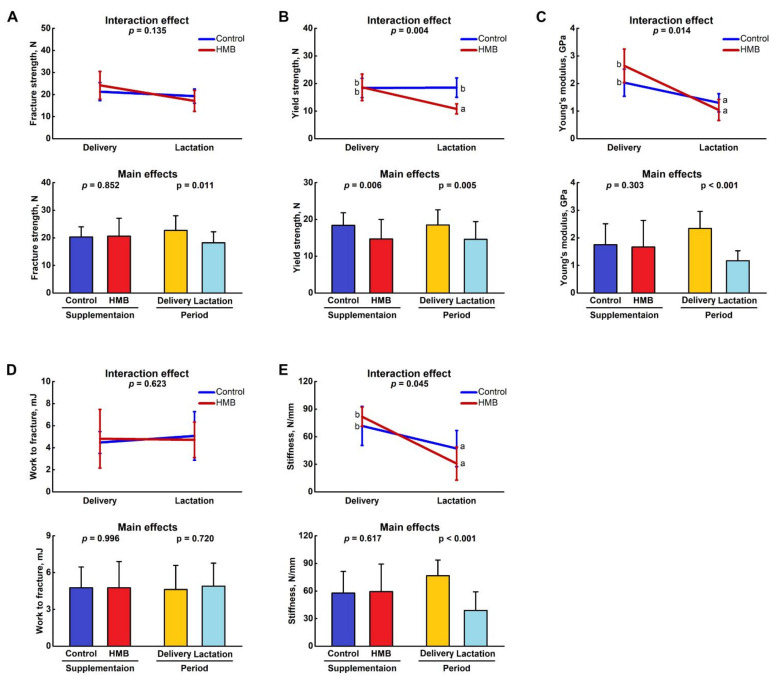
Effect of HMB supplementation (0.02 g/kg b.w.) during the middle trimester of pregnancy (13–26 d) on (**A**) fracture strength, (**B**) yield strength, (**C**) Young’s modulus, (**D**) work to fracture and (**E**) stiffness of femora of spiny mice at delivery or after the lactation period. Figure shows LSMeans ± standard deviation as well as *p*-values of a two-way ANOVA with the supplementation (control, HMB) and period (delivery, lactation) and their interaction as fixed effects. Means with different letters in interaction effect plots differ significantly (Tukey’s post-hoc test).

Figure 3 shows the mechanical parameters of the femora from the control and HMB-supplemented females. HMB given during middle pregnancy did not influence the value of the ultimate fracture strength, but was affected by the period, and lactating dams had femora which were characterized by the lower ultimate fracture strength (*p* = 0.011; Figure 3A). There was a significant interaction of supplementation and period on the femoral yield strength and the HMB treatment led to a significantly decreased value in lactating dams compared to other groups (*p* = 0.004; Figure 3B). Young’s modulus and stiffness was dependent on the period; the lowest values were in groups of lactating dams irrespective of the treatment (*p* < 0.001 in both cases; Figure 3C,E, respectively). No differences were noted in the value of the work to fracture between the groups (Figure 3D).

**Figure 4:**
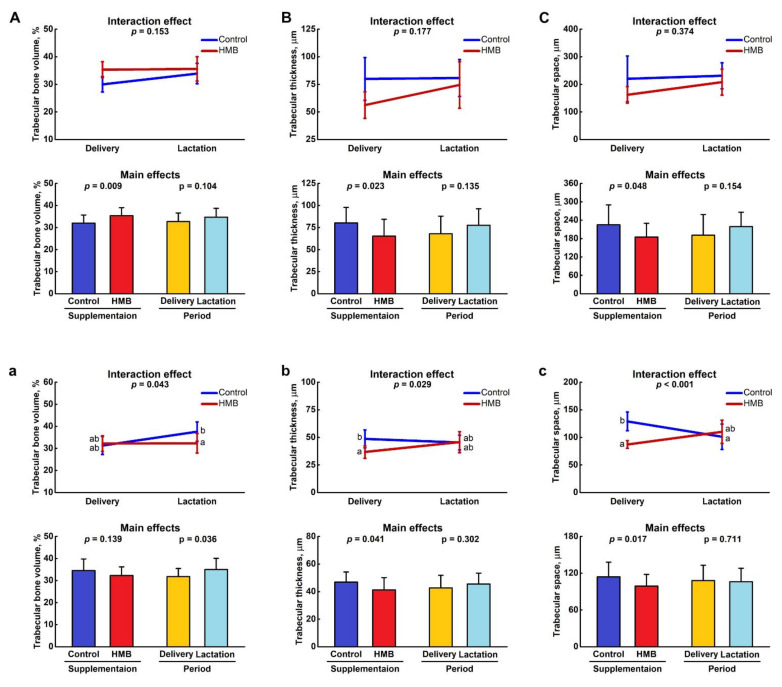
Effect of HMB supplementation (0.02 g/kg b.w.) during the middle trimester of pregnancy (13–26 d) on histomorphometry of femoral trabeculae in bone (**A**–**C**) metaphysis and (**a**–**c**) epiphysis of femora of spiny mice at delivery or after the lactation period. Figure shows LSMeans ± standard deviation as well as *p*-values of a two-way ANOVA with the supplementation (control, HMB) and period (delivery, lactation) and their interaction as fixed effects. Means with different letters in interaction effect plots differ significantly (Tukey’s post-hoc test).

Figure 4 shows the histomorphometry of spiny mice femora distal metaphysis (Figure 4A–C) and epiphysis (Figure 4a–c). All determined histomorphometrical parameters of the metaphyseal trabeculae were dependent on the HMB supplementation, and the increase of trabecular bone volume was observed (*p* = 0.009; Figure 4A) which resulted from the decrease of trabecular space (*p* = 0.048; Figure 4C), rather than from the increase of trabecular thickness (*p* = 0.023; Figure 4B). The trabecular bone volume in femoral epiphysis was dependent on the period, and higher value was noted in the control lactating dams compared to the value noted in the HMB-treated lactating dams (*p* = 0.043; Figure 4a). These values did not differ from that noted in groups of delivered dams irrespective of the treatment. The trabecular thickness was affected by the HMB treatment at delivery time (*p* = 0.029; Figure 4b). Trabecular space was the highest in the delivered dam controls, while it was the lowest in the lactating controls and HMB-treated delivered dams. The trabecular space in the HMB-treated lactating dams did not differ from values determined in other groups (*p* < 0.001; Figure 4c). Figure 2A shows representative µCT images of the sagittal sections of the femur distal diaphysis in the examined groups.

### 3.2. OPG, OC, BMP-2, TIMP-2, MMP-8 and MMP-13 Immunoreaction in Growth Plate Cartilage

**Figure 5:**
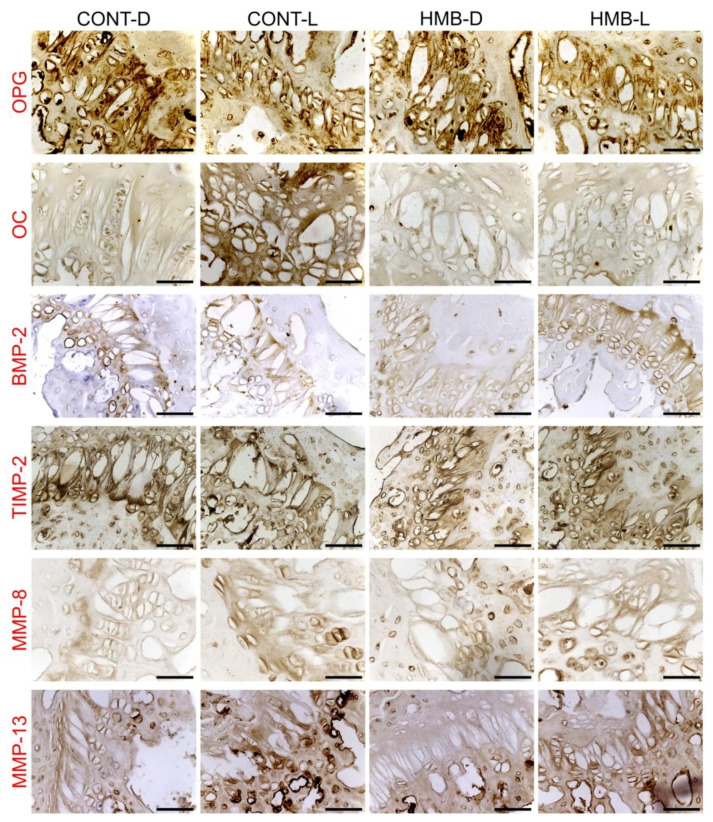
Representative images of the immunohistochemical reactions for osteoprotegerin (OPG), osteocalcin (OC), bone morphogenetic protein 2 (BMP-2), tissue inhibitor of metalloproteinases 2 (TIMP-2), matrix metalloproteinase 8 (MMP-8) and matrix metalloproteinase 13 (MMP-13) in the growth plate cartilage of femora from pregnant female controls (not receiving HMB) at delivery (CONT-D) or after the lactation period (CONT-L) and from pregnant HMB females (receiving HMB at a dose of 0.02 g/kg b.w. during the middle trimester of pregnancy) at delivery (HMB-D) or after the lactation period (HMB-L). All the scale bars represent 40 µm.

Figure 5 shows representative images of the immunohistochemical reactions for OPG, OC, BMP-2, TIMP-2, MMP-8 and MMP-13 in the growth plate cartilage; the results of analyses of the intensity of immunoreactions are presented in Supplementary Table S1. HMB supplementation did not influence the intensity of immunoreaction of TIMP-2 and BMP-2 in growth plate cartilage. The intensity of the immunoreaction of TIMP-2 was strongest in the CONT-D group and differed from that noted in the lactating dams, irrespective of HMB supplementation; while a weaker TIMP-2 reaction was observed in the CONT-L group, which differed significantly from the intensity of immunoreaction observed in the HMB-D group (*p* = 0.047). The CONT-D and HMB-L groups showed stronger intensity of reaction of BMP-2 in comparison to the other two groups (*p* < 0.001). The intensity of OPG reaction was dependent both on the period (*p* < 0.001), with a stronger reaction observed after delivery and the supplementation with a slightly weaker reaction observed in the HMB-treated dams (*p* = 0.047). OC immunoreaction was dependent on both HMB supplementation and the period, with the strongest OC immune reaction observed in the CONT-L group and similar, much weaker reactions observed in the other groups (*p* < 0.001). MMP-13 immunoreaction was significantly stronger in the CONT-L group compared to that of the other groups (*p* < 0.001). MMP-8 immunoreaction was stronger in the HMB supplemented dams (*p* < 0.001), and lactating dams, irrespective of treatment (*p* < 0.001).

### 3.3. OPG, OC, BMP-2, TIMP-2, MMP-8 and MMP-13 Immunoreaction in Trabecular Bone

**Figure 6:**
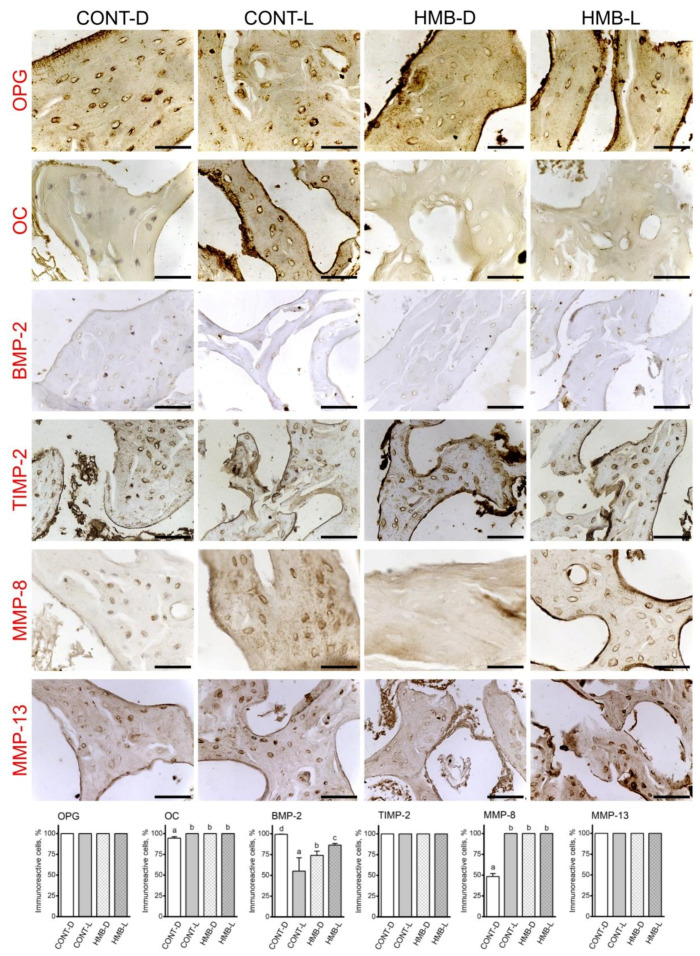
Representative images of the immunohistochemical reactions and the percent of immunoreactive cells for osteoprotegerin (OPG), osteocalcin (OC), bone morphogenetic protein 2 (BMP-2), tissue inhibitor of metalloproteinases 2 (TIMP-2), matrix metalloproteinase 8 (MMP-8) and matrix metalloproteinase 13 (MMP-13) in the trabecular bone of femora from pregnant female controls (not receiving HMB) at delivery (CONT-D) or after the lactation period (CONT-L) and from pregnant HMB females (receiving HMB at a dose of 0.02 g/kg b.w. during the middle trimester of pregnancy) at delivery (HMB-D) or after the lactation period (HMB-L). All the scale bars represent 40 µm. Bar plots show the percentage of immunoreactive osteocytes in the trabecular bone in each group.

Figure 6 shows representative images of the immunohistochemical reactions for OPG, OC, BMP-2, TIMP-2, MMP-8 and MMP-13 in trabecular bone; the results of analyses of the intensity of immunoreactions are presented in Supplementary Table S2. HMB supplementation had no effect on the number of BMP-2 immunoreactive bone cells in trabecular bone (Figure 6). The highest number of immunoreactive BMP-2 cells was observed in the CONT-D group, with lower numbers observed in the HMB supplemented group, and the lowest number of immunoreactive cells in the CONT-L group. All groups significantly differed from one other. In the case of OPG, TIMP-2 and MMP-13, all cells showed a positive reaction. OC immunoreaction was noted in a significantly lower number of bone cells in the CONT-D group, compared to that of the other groups, in which all cells showed the reaction. The number of cells showing immune reactivity for MMP-8 was significantly lower, by almost half in the CONT-D group, compared to that of the other groups, which were not significantly different from one another (Figure 6).

Analysis of the OPG immunoreaction in trabecular bone showed (Supplementary Table S2) the strongest reaction in the CONT-D group which was significantly stronger than in the CONT-L and HMB-D groups (*p* = 0.026). The reaction in the CONT-D group was mainly detected in the periterritorial zone of cells. The intensity of OPG reaction in the CONT-L group was from low to moderate in the periterritorial zone of cells. Most cells in the HMB-D group displayed a very low OPG signal, with only a few cells having a strong reaction in the periterritorial zone. Bone cells in the HMB-L group displayed varied reactions ranging from a low to very strong reaction in both the cytoplasm and periterritorial zone. The OPG immunoreaction in the matrix was strong in the HMB-D group, and low with similar intensities in the other groups.

The OC immune reaction was dependent on the HMB supplementation and period (*p* < 0.001). In general, HMB supplementation led to the decrease of the intensity of the OC reaction in trabecular bone irrespective of the period detection. The significantly strongest reaction was detected in the CONT-L group compared to the other groups. OC immunoreaction was detected mainly in bone cells from the CONT-D group, with only a very scant number of cells with well-marked reactions in the periterritorial zone. Quite the opposite situation was observed in the CONT-L group, where bone cells had a very strongly marked reaction in the periterritorial zone (Figure 6). Additionally, the intensity for the OC signal was very visible in the matrix of the CONT-L group. The HMB supplementation led to weak OC immunoreaction with a similar intensity in all cells. Only very few cells displayed a moderate signal for OC in the HMB-L group. The HMB treatment results also showed a weak signal for OC in the matrix.

The BMP-2 immunostaining was strongest in the lactating dams, irrespective of treatment, although the HMB supplementation decreased the intensity of the BMP-2 signal in delivered dams (HMB-D), which was significantly weaker compared to that observed in the other groups, including the CONT-D group, where the BMP-2 signal was moderate (*p* < 0.001). BMP-2 immunoreaction in the matrix was well-marked in both lactating groups (with signals of similar intensities), while the intensity of the BMP-2 reaction was low in both groups of dams delivered only.

The intensity of TIMP-2 immunoreaction was higher in the HMB- D group compared to other groups, where the intensity did not differ between them (*p* = 0.002). A strong TIMP-2 signal was detected in all cells within the periterritorial zone. Well-marked and strong TIMP-2 immunoreaction was observed in the matrix of the HMB-D group, while no strong reaction was observed in the other groups.

MMP-8 immunoreaction was dependent on both the HMB supplementation and the period, and was weaker following HMB supplementation, and stronger in lactating dams, irrespective of the treatment (*p* < 0.001). The MMP-8 immunoreaction showed a positive signal of differing intensities, ranging from very weak to moderate in the CONT-D group. The MMP-8 positive signal was well-marked in the CONT-L group. The MMP-8 positive signal was similar and of low intensity in the HMB-D group, while the reaction was strong in the periterritorial zone of cells in the HMB-L group. Detection of MMP-8 immunoreaction in the matrix showed that HMB supplementation during pregnancy led to a low intensity of MMP-8 immune reaction during lactation, while just after delivery this positive signal was of high intensity.

The intensity for MMP-13 immune reaction was dependent on the HMB supplementation and period (*p* < 0.001). MMP-13 immunoreaction was higher in lactating dams compared to delivered only dams, and stronger after HMB supplementation. The strongest MMP-13 signal was observed in the HMB-L group, a lower signal observed in the CONT-L group, and the lowest signal observed in both groups of delivered only dams. The MMP-13 signal in the HMB-L group was very strong in most bone cells. The majority of cells in the CONT-L group displayed MMP-13 immunoreaction of varying intensities, ranging from weak to very strong signals. The immune reaction in the CONT-D group ranged from weak to moderate. Finally, the MMP-13 immunoreaction in the HMB-D group ranged from low to well-marked intensities. The intensity of matrix MMP-13 immunoreaction was strong in the HMB supplemented dams, irrespective of the period of detection.

### 3.4. OPG, OC, BMP-2, TIMP-2, MMP-8 and MMP-13 Immunoreaction in Compact Bone

**Figure 7:**
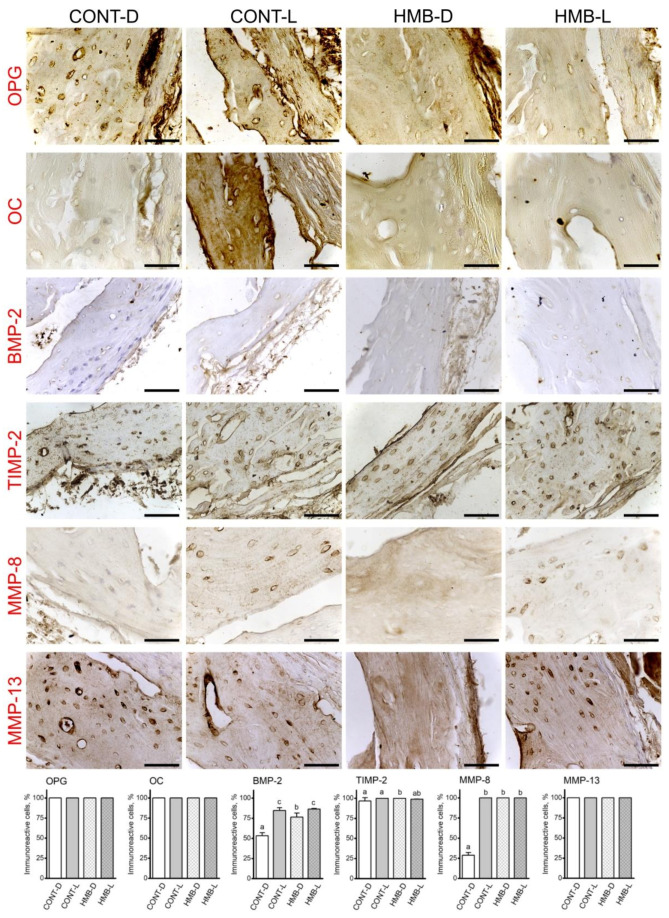
Representative images of the immunohistochemical reactions and the percent of immunoreactive cells for osteoprotegerin (OPG), osteocalcin (OC), bone morphogenetic protein 2 (BMP-2), tissue inhibitor of metalloproteinases 2 (TIMP-2), matrix metalloproteinase 8 (MMP-8) and matrix metalloproteinase 13 (MMP-13) in the compact bone of femora from pregnant female controls (not receiving HMB) at delivery (CONT-D) or after the lactation period (CONT-L) and from pregnant HMB females (receiving HMB at a dose of 0.02 g/kg b.w. during the middle trimester of pregnancy) at delivery (HMB-D) or after the lactation period (HMB-L). All the scale bars represent 40 µm. Bar plots show the percentage of immunoreactive osteocytes in the trabecular bone in each group.

Figure 7 shows representative images of the immunohistochemical reactions for OPG, OC, BMP-2, TIMP-2, MMP-8 and MMP-13 in compact bone; the results of analyses of the intensity of immunoreactions are presented in Supplementary Table S3. HMB supplementation had no effect on the number of OPG, OC, TIMP-2 and MMP-13 immunoreactive cells in compact bone. The highest number of immunoreactive BMP-2 cells was observed in the CONT-L and HMB-L groups, with a lower number of immunoreactive cells in the HMB-D group, and the lowest number of immunoreactive cells in the CONT-D group. The number of cells displaying MMP-8 reactivity was lowest in the CONT-D group compared to the other groups, which did not differ from one another (Figure 7).

The intensity of OPG immunoreaction in compact bone was influenced by the interaction of the HMB treatment and period (*p* < 0.001; Supplementary Table S3). The strongest OPG immunoreaction was observed in both control groups, with lower immunoreaction in the HMB-D group, and the lowest in the HMB-L group. In the CONT-D group, a very strong reaction of similar intensity was detected in almost all cells in the periterritorial zone. The OPG immunoreaction in the CONT-L group was similar in all cells, with a very strong or moderate immunoreaction in the periterritorial zone. The majority of cells in the HMB-D group displayed a moderate OPG positive signal, with only a few cells having a poorly-marked reaction in the periterritorial zone. Cells in the HMB-L group displayed a varying signal, ranging from low to moderate in the periterritorial zone. The OPG immune reaction in the matrix was strong in the control and HMB-D groups, while a low intensity signal was observed in the HMB-L group.

The OC immune reaction was dependent on the HMB supplementation and period (*p* < 0.001). The significantly strongest reaction was detected in the CONT-L group, lower in the HMB-D group, and the lowest in both the CONT-D and HMB-L groups. Cells in the CONT-L group had a strong OC signal detected in the cytoplasm and in the periterritorial zone. Quite the opposite situation was observed in the CONT-D group, where cells had a weakly marked periterritorial zone. In the HMB-L group only a few cells showed well-marked reactions in the periterritorial zone. HMB supplementation led to well-marked OC immunoreaction in the periterritorial zone and with a similar intensity in almost all the cells in only delivered dams. The strong OC immunoreaction was noted in the CONT-L group in the matrix, while it was weak in the other groups.

The intensity for BMP-2 immunoreaction in the HMB-L group was the strongest compared to the other groups (*p* < 0.001; Supplementary Table S3, Figure 7), where the reaction ranged from weak to moderate in the periterritorial zone, with very few cells showing a well-marked, moderate reaction in the cytoplasm. The immunoreaction in the HMB-D group was low or very poorly-marked in the cytoplasm, and weak in the periterritorial zone. BMP-2 immunoreaction in the matrix of all groups was similar.

TIMP-2 immunoreaction was very strong and well-marked in the periterritorial zone, and moderate in the cytoplasm of all groups. However, the weakest intensity for the TIMP-2 reaction was detected in the HMB-D group, compared to that observed in the other groups, where the reaction was similar (*p* = 0.007).

The MMP-8 immune reaction was dependent on the HMB supplementation and period (*p* = 0.016 and *p* < 0.001, respectively, Supplementary Table S3). The stronger reaction was detected in the control groups, and lower intensity was detected in delivered dams. In general, in lactating dams, the immune reaction was well-marked in many cells; however, there were cells with moderate or weak positive reactions, mainly in the periterritorial zone (Figure 7). MMP-8 immunoreaction in the CONT-D group ranged from moderate to weak, while it was well-marked in the HMB-D group. The intensity of MMP-8 immunoreaction in the matrix was strong in the HMB-D group, and weak in the other groups.

The strongest MMP-13 positive reaction was detected in the HMB-L group, where most cells displayed a strong signal in the periterritorial zone and only a few cells displayed a moderate positive reaction. MMP-13 immunoreaction in the CONT-D group was lower from that observed in the HMB-L group, but higher compared to the other two groups (*p* < 0.001, Supplementary Table S3). MMP-13 immunoreaction in cells in the HMB-D group ranged from weak to moderate, in the periterritorial zone it was weak and not well-marked or moderate and well-marked. The intensity of MMP-13 immunoreaction in the matrix was strong in the HMB-L group, while in other groups was it low and similar.

### 3.5. OPG, OC, BMP-2, TIMP-2, MMP-8 and MMP-13 Immunoreaction in Articular Cartilage

**Figure 8:**
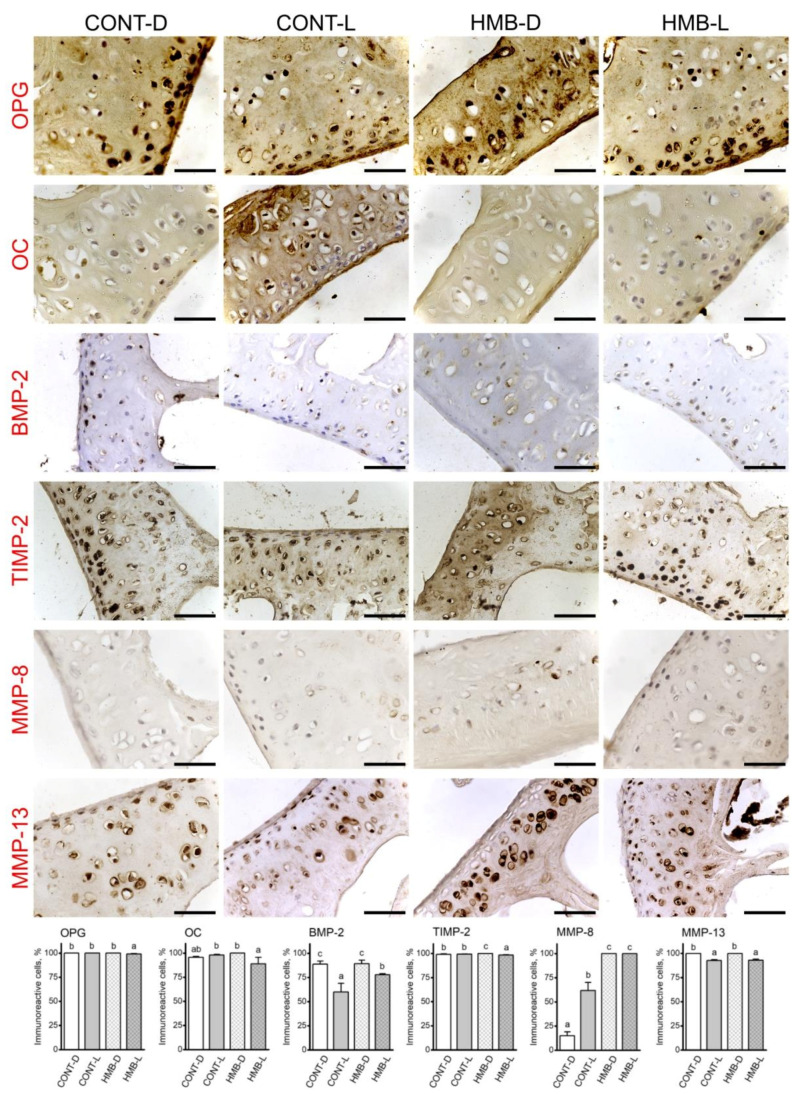
Representative images of the immunohistochemical reactions and the percent of immunoreactive cells for osteoprotegerin (OPG), osteocalcin (OC), bone morphogenetic protein 2 (BMP-2), tissue inhibitor of metalloproteinases 2 (TIMP-2), matrix metalloproteinase 8 (MMP-8) and matrix metalloproteinase 13 (MMP-13) in the articular cartilage of femora from pregnant female controls (not receiving HMB) at delivery (CONT-D) or after the lactation period (CONT-L) and from pregnant HMB females (receiving HMB at a dose of 0.02 g/kg b.w. during the middle trimester of pregnancy) at delivery (HMB-D) or after the lactation period (HMB-L). All the scale bars represent 40 µm. Bar plots show percentage of immunoreactive chondrocytes in the articular cartilage in each group.

Figure 8 shows representative images of the immunohistochemical reactions for OPG, OC, BMP-2, TIMP-2, MMP-8 and MMP-13 in the articular cartilage; the results of analyses of the intensity of immunoreactions are presented in Supplementary Table S4. HMB supplementation had no effect on the number of OC, TIMP-2 and MMP-13 immunoreactive osteocytes in articular cartilage; however, there was a significant influence of the events linked with pregnancy alone or pregnancy and lactation together (Figure 8).

The number of OPG immune reactive cells was slightly lower in the HMB-L group compared to the other groups, where a positive reaction was detected in all cells. The number of OC reactive cells was highest in the CONT-L and HMB-D groups and did not differ from the number of cells counted in the CONT-D group, while the lowest number of OC positive cells was detected in the HMB-L group.

The highest number of TIMP-2 positive cells was detected in the HMB-D group, with lower numbers in both control groups and the lowest number of TIMP-2 positive cells in the HMB-L group. Moreover, the number of BMP-2 positive cells was highest in both groups of delivered only dams irrespective of treatment, lower in the HMB-L group, and the lowest in the CONT-L group. The number of MMP-13 positive cells was higher in delivered only dams and lower in the lactating dams, irrespective of treatment. The highest number of MMP-8 positive cells was detected in the HMB treated groups, irrespective of the period of determination, while the number of MMP-8 positive cells was lower in the lactating control dams, and the lowest in the delivered control dams (Figure 8).

OPG immunostaining in articular cartilage showed a strong positive cytoplasmic signal in all groups except the CONT-L group, where the positive signal was weaker (*p* = 0.004, Supplementary Table S4). The strongest positive reaction for OC was observed in the CONT-L group, while lower signal was in the CONT-D group, and the lowest in both HMB treated groups of dams (*p* = 0.004). TIMP-2 reaction was dependent on both supplementation and period (*p* < 0.004 and *p* = 0.006, respectively, Supplementary Table S4). A strong positive reaction for TIMP-2 was observed in delivered only dams, with a lower signal observed in the lactating dams, irrespective of the treatment. In the case of BMP-2, the strongest reaction was observed in the HMB-L group, with a lower signal in the CONT-D group, and the lowest in the other two groups (*p* < 0.001, Supplementary Table S4). Furthermore, the strongest positive MMP-13 reaction was observed in the CONT-D and HMB-L groups, with a lower reaction observed in the HMB-D group, and the lowest in the CONT-L group (*p* = 0.001, Supplementary Table S4). The MMP-8 positive signal was strongest in both HMB=treated groups of dams and lower in the CONT-L group, and the lowest in the CONT-D group (*p* = 0.005, Supplementary Table S4, Figure 8).

## 4. Discussion

Bone is a metabolically active tissue which undergoes a continuous remodeling process throughout an individual’s lifetime. Peak bone mass is reached at a certain age, after which the process of remodeling occurs at a slower rate, with bone-forming processes slowing down, and a gradual loss of bone mass begins [1,2,3,48]. The processes involved in bone tissue remodeling, irrespective of sex, are strictly controlled by many different factors including hormones and nutrition. The main sex hormones, like estrogen in women, play an important role in the regulation of bone metabolism. Additionally, the levels of these hormones fluctuate significantly throughout life, and important events linked with a decrease in estrogen are pregnancy and aging. These events significantly affect the rate of bone turnover, which lead to osteopenia and osteoporosis, and finally to bone fractures. Osteoporosis is more commonly associated with aging; however, it has been proven that increased bone loss occurs in women after pregnancy and lactation [1,2,3,49,50]. Pregnancy and lactation are physiological states which occur during a woman’s lifetime; however, both are very specific. During both these periods, the demand for calcium increases [4,5,6]. It is suggested by some clinicians that the increase in calcium absorption from the intestine is not sufficient for the pregnant woman and the developing skeletal system of the fetus. For this reason, an increase in maternal bone resorption and bone loss can also be observed during pregnancy. The same problem occurs during the lactation period and is also generally associated with pregnancy and lactation-associated osteoporosis (PLO) [51,52]. The resulting severe back pain in the young mother affects basic life functions. The first signs of PLO, besides the pain, include worsening thoracic kyphosis and a reduction in height [9,10,11]. As a consequence of PLO, low-energy vertebral fractures are noted [53,54,55].

There is no data concerning HMB supplementation in pregnant women or information concerning the effect of HMB use, due to the lack of recommendation for HMB supplementation during pregnancy. Available data from animal experiments have showed different effects of HMB supplementation, which can be species-dependent [21,40,56,57,58]. However, there is still a dearth of studies related to bone metabolism in pregnant females supplemented with HMB. Our recent model study showed that HMB supplementation during pregnancy intensifies bone formation processes and prevents bone loss during pregnancy in mice [27]. All these recently observed changes in bone properties are dependent on the intensification of bone turnover, which depends on the activity of the osteoblasts and osteoclasts [59,60]. The main factors regulating this process are osteoprotegerin (OPG), an osteoclastogenesis inhibitory factor released by osteoblasts; RANKL, a glycoprotein produced by mature osteoblasts and their precursors, that activate the process of creating mature osteoclasts; as well as a RANK glycoprotein present on the precursors of osteoclasts. As a result of RANK activation by RANKL, the maturing osteoclast undergoes structural and metabolic changes that initiate a resorptive effect. OPG can bind to RANKL, since it is a soluble receptor, which then prevents RANKL from binding to RANK, and consequently stops the osteoclast maturation pathway. The differentiation, maturation and metabolic activity of osteoclasts, and thus, the intensity of bone resorption, are dependent on the relative balance between the levels of RANKL and OPG. When RANKL is superior to OPG, the rate of bone tissue resorption is pathologically increased, and when OPG is superior to RANKL, the intensity of the bone loss process is pathologically reduced [31].

Our study showed a limited and period-dependent effect of the HMB supplementation on compact bone which forms the bone mid-diaphysis. The positive effect of the HMB treatment was evident only in bone cross-sectional geometry (mean relative wall thickness and cortical index) at the delivery period. Bone geometry reflects not only the size but also the bone shape. Our results showed that there was a loss of bone mass during lactation, which could indicate the significant demand for minerals for milk synthesis. However, there no statistically significant alterations observed in bone geometry. Bones after lactation become weaker and more prone to fractures at weaning, irrespective of the treatment, as shown by decreased ultimate fracture strength and Young’s modulus. Both these parameters are dependent on bone mineralization [61] and the decrease of bone strength is in agreement with the observed bone mass loss after lactation. However, the supplementation-dependent changes in the values of bone mechanical parameters are not strictly dependent on the bone mineral phase, such as yield strength and stiffness [62], but are more related to the bone organic compartment, which can suggest more intensive bone turnover in the HMB-treated dams compared to the control ones.

This effect of HBM treatment was also observed in the changes of trabecular bone microarchitecture. In general, trabecular bone is characterized with a higher bone remodeling rate than compact bone and, therefore, any changes are firstly observed in the histomorphometry of trabeculae [63]. Our study showed the positive effect of HMB supplementation in the metaphysis irrespective of the period while in epiphysis. This positive effect was especially evident in dams at delivery time when compared to the non-supplemented control. All results discussed above might suggest that HMB should be given for a longer time and during lactation, rather than only for a limited time during mid-pregnancy. Spiny mice are characterized by the longest duration of pregnancy among other laboratory rodents, but it is still shorter compared to human pregnancy; thus, the bone rate metabolism can differ compared to that in humans. For this reason, further studies are needed, including other animal models.

Immunohistochemical analysis of femora from the spiny mice in the current study showed that HMB supplementation during pregnancy influenced the level of certain non-collagen proteins in the bone tissue. Femora from the group of the controls only delivered females (no HMB supplementation) had the most visible immunohistochemical reactions, confirming increased intensity for OC and OPG. Both proteins are responsible for bone mineralization, and osteocalcin is considered a highly specific late marker of bone turnover. OPG inhibits the activity of osteoclasts, and thus, protects the bones against the loss of their minerals, and osteocalcin facilitates bone mineralization processes by binding calcium and hydroxyapatites [64].

OPG was intensively produced during pregnancy in our control dams. The increased OPG signal can likely indicate the increase of OPG synthesis, which probably protects the mother‘s bones against intensive loss of tissue, which can lead to poorly mineralized bones in the fetus. The opposite effect was observed in the group of HMB-treated pregnant females, in whom a significantly lower intensity of OPG reaction was noted. Previously, it has been proven that dietary HMB supplementation during pregnancy enhances bone mineralization in offspring [14,17]. Until the current study, there was no available data concerning the effects of HMB supplementation on the bone tissue of dams following pregnancy and the lactation period when their offspring are weaned. Data from the current study indicated that the recovery of the maternal skeleton begins upon weaning. These results also showed that the trabecular bone, which is characterized by faster bone turnover compared to compact bone (trabecular bone is lost at a rate of between 1% and 3% per month during lactation in humans [8]), is more strongly protected against bone loss than compact bone, through the increased expression of OPG. Cohen et al. [8] suggest that in PLO women, a functional osteoblast defect occurs, which can be reflected by the OPG decrease. Moreover, OC is a protein responsible for bone mineralization, but its overproduction also indicates the intense bone processes related to osteoporosis. Neither HMB supplementation, nor pregnancy, significantly influenced OC immune reaction in the mice in the current study. However, considering that the OPG reaction was decreased in control dams at weaning and the intensity of the OC immune reaction was strongly increased, it can indicate that there is very intensive bone turnover. The opposite effect was observed in the HMB-treated pregnant females, in whom a significantly lower reaction for OC was noted at delivery.

These observations are in agreement with a study showing a slight increase in osteocalcin and other bone turnover markers like alkaline phosphatase and the C-telopeptide of type I collagen, which was linked with the healing of fresh bone fractures in PLO women [53,65,66]. Bone biopsies from PLO women also have proven enhanced resorption or decreased activity of osteoblasts [8,10,67].

The intensity of the BMP-2 signals, a protein related to the osteoinduction potential of the bone and the regulation of the growth of chondrocytes in the cartilage plate [68,69], was highest in the pregnant female controls. At weaning, a decrease in BMP-2 was noted, which was especially visible in the HMB-treated dams in trabecular bone. The decreased BMP-2 signal observed in the HMB-treated dams was probably related to the OPG reaction, and the reduced activity of osteoclasts, resulting in improved bone quality [27].

It is well known that BMP-2 is expressed in both osteocytes and osteoblasts [70], and if OPG expression increases, it can be suggested that the number of mature osteoclasts is decreased, which in turn would lead to decreased BMP-2 expression. BMP-2 regulates OPG mRNA levels in osteoblasts, increasing the RANKL/OPG ratio and promoting osteoclastogenesis [71].

The intensity of the TIMP-2 immune reaction, a natural inhibitor of the matrix metalloproteinases, a group of peptidases involved in the degradation of the extracellular matrix, was highest in the group of HMB-treated pregnant females, with the TIMP-2 reaction decreasing upon weaning. The opposite reaction was observed in compact bone following HMB supplementation. The TIMP-2 immune reaction in trabecular bone was relatively high in the control dams; however, in compact bone, the intensity of the TIMP-2 reaction decreased compared to the time of delivery. On the other hand, HMB supplementation significantly increased the intensity of the TIMP-2 reaction decrease in compact bone, indicating different bone turnover processes in these two bone tissues. During a physiological pregnancy, in its final stage, the growth and development of the fetus is nearly complete and the metabolic processes directed towards the reconstruction of the maternal bone tissue have not yet intensified. TIMP-2 is responsible for the stimulation of osteoclastic bone resorption, and it is also associated with bone matrix mineralization [72,73]. In the case of HMB-induced dams, the increase of the OPG immune reaction, which is associated with decreased BMP-2, the increase in the TIMP-2 reaction at delivery was not able to increase bone resorption, which was similar to what was observed in delivered dam controls in a previous study [27].

This observation of the immunoreaction of different bone proteins involved in bone metabolism is supported by the result of the mechanical testing. It seemed that HMB supplementation increasing bone turnover allowed for higher calcium transfer taking into account the bone weight in the current study and changes in bone in a previous study [27] However, it should be emphasized that it can be dependent on the model species, because different effects were observed in swine or mink offspring delivered by the HMB-treated mothers. Current studies also showed that significant changes are observed in the compact bone, which became more susceptible to fracture, against which HMB-treatment did not prevent. The opposite effect was observed in trabecular bone, where significant improvement of the histomorphometry was noted after HMB supplementation taking into account the bone volume and trabecular space and number.

In the current study we also investigated two MMPs, which remodel collagen present in the bone extracellular matrix, and participate in the degradation and regeneration of the surrounding chondrocytes or osteocytes extracellular matrix [32]. The results obtained showed that the reaction for MMP-13 was lower during pregnancy, and was dependent on the region of determination, with a lower reaction in trabecular bone and a higher reaction in compact bone. HMB supplementation decreased cell activity mainly during lactation. The opposite effect was observed in the matrix, where MMP-13 reaction was very strong in the control dams, and following HMB supplementation, a further increase was observed. Moreover, HMB supplementation significantly increased the MMP-8 reaction in trabecular bone during pregnancy, which then decreased at weaning. The opposite effect was observed in the control group. Compact bone cells in the control group expressed MMP-8 intensively during pregnancy. Both MMP-13 and MMP-8 function as enzymes. Their primary function is the degradation of type I, II, III and IV collagens in the matrix during the development and remodeling of bone and cartilage [32].

Furthermore, the immunohistochemical analysis of articular cartilage showed that HMB-treatment reduced the reaction for osteocalcin irrespective of the period, while the OC reaction in the pregnant female controls was very high. Under certain conditions, chondrocytes can alter their activity and express bone-related proteins such as osteocalcin, changing the composition of the matrix and leading to accelerated degradation. In general, chondrocytes, which synthesize type II and type IV collagen under normal physiological circumstances, have a stable phenotype in mature adult articular cartilage [74]. OPG intensity was decreased after lactation compared to that observed during pregnancy in the control dams. The decrease in OPG immune reaction was not observed in HMB-treated dams. In general, HMB supplementation increased the reaction of all the investigated proteins in articular cartilage. Pregnancy is characterized by an increase in the BMP-2 immune reaction compared to that observed during the lactation period. A significant increase in MMP-8 and decrease in MMP-13 was observed during lactation compared to that observed in pregnancy. MMPs play the same role in articular cartilage as that in bone tissue. BMP-2, however, plays an opposite role in articular cartilage compared to its role in bone tissue, and protects against damage. Articular cartilage homeostasis depends on the balance between aggrecan and collagen content. Their imbalance leads to the loss of the structural integrity of articular cartilage, which as well as the natural lack of blood and lymph supply, and its neural character, leads to degradation.

All the results from the current study are in agreement with a recent study, which shows that HMB supplementation intensifies bone and matrix turnover, which is proven by the analysis of the content of immature collagen. The previous study also shows that HMB enhances the content of proteoglycans in the articular cartilage in pregnant females [27]. Chondrocytes in articular cartilage produce not only type II collagen, but also proteoglycans, synthesized and released into the matrix by chondrocytes, and their concentration indicates their metabolic ability [75].

We agree that the present study has some limitations. No bone turnover markers (osteocalcin, bone alkaline phosphatase, procollagen type I amino-terminal propeptide, collagen type I cross-linked c-terminal telopeptide, pyridinoline, deoxypyridinoline) or BMD and bone calcium content in blood serum were investigated. Moreover, no Western blots were performed for the determination of the presented proteins, and no PCR was conducted for their gene expression or changes following HMB supplementation. Therefore, further studies are needed in this area. Furthermore, future studies should also include the determination of the differentiation between osteoblasts, osteoclasts, chondrocytes and osteocytes as well as immunolocalization of nuclear factor kappa-Β ligand (RANKL), which are missing in presented study.

## 5. Conclusions

The number of studies involving HMB supplementation in relation to pregnancy has increased over the past few years. The current study has now also provided data concerning the influence of HMB supplementation during pregnancy on maternal bone structure postpartum and during weaning. Based on the results of the current study, it can be assumed that the administration of HMB to pregnant females has a very positive effect on articular cartilage and bone. This protective effect before bone tissue loss was more visible in trabecular bone than compact. However, the possible preventative effect of HMB supplementation before PLO in humans needs to be further studied.

## Data Availability

The data presented in this study are available upon request from the corresponding author.

## Supplementary Materials

The following are available online at www.mdpi.com/article/10.3390/jcm10214808/s1, Figure S1. Representative pictures of the antibody control for all immunohistochemical reactions developed in 3,3’-diaminobenzidine tetrahydrochloride (DAB); counterstaining performed with Mayer’s hematoxylin. All the scale bars represent 50 μm. Table S1. Effect of HMB supplementation (0.02 g/kg b.w.) during the middle trimester of pregnancy (13-26 d) on the intensity of immunoreactions (pixel value) against bone turnover proteins in the growth plate cartilage of spiny mice femora. Table S2. Effect of HMB supplementation (0.02 g/kg b.w.) during the middle trimester of pregnancy (13-26 d) on the intensity of immunoreactions (pixel value) against bone turnover proteins in the trabecular bone of spiny mice femora. Table S3. Effect of HMB supplementation (0.02 g/kg b.w.) during the middle trimester of pregnancy (13-26 d) on the intensity of immunoreactions (pixel value) against bone turnover proteins in the compact bone of spiny mice femora. Table S4. Effect of HMB supplementation (0.02 g/kg b.w.) during the middle trimester of pregnancy (13-26 d) on the intensity of immunoreactions (pixel value) against bone turnover proteins in the articular cartilage of spiny mice femora.
