# Supplementary material for: Femoral µCT Analysis, Mechanical Testing and Immunolocalization of Bone Proteins in β-Hydroxy β-Methylbutyrate (HMB) Supplemented Spiny Mouse in a Model of Pregnancy and Lactation-Associated Osteoporosis
Source: J Clin Med. 2021 Oct 20;10(21):4808. doi: 10.3390/jcm10214808 (PMC8584851; doi:10.3390/jcm10214808)
Supplement: Supplementary file 1 [file jcm-10-04808-s001.zip › jcm-1402166-supplementary.pdf]

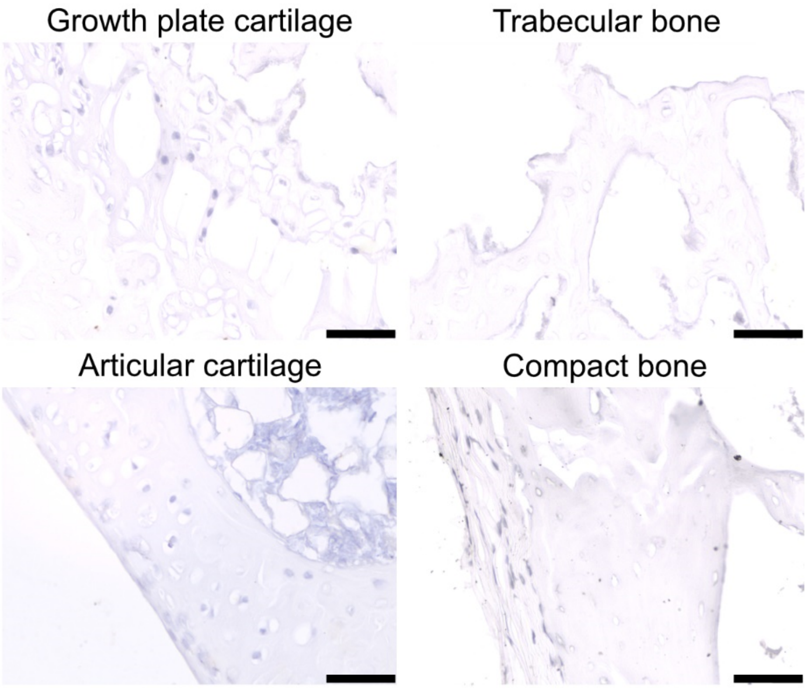

Figure S1. Representative pictures of the antibody control for all immunohistochemical reactions developed in 3,3′-diaminobenzidine tetrahydrochloride (DAB); counterstaining performed with Mayer’s hematoxylin. All the scale bars represent 50 μm.

Table S1. Effect of HMB supplementation (0.02 g/kg b.w.) during the middle trimester of pregnancy (13–26 d) on the intensity of immunoreactions (pixel value) against bone turnover proteins in the growth plate cartilage of spiny mice femora.

**Treatment**	**OPG**	**OC**	**BMP-2**	**TIMP-2**	**MMP-8**	**MMP-13**
Main effect supplementation						
Control	127 ± 12	93 ± 29	62 ± 20	110 ± 15	85 ± 13	77 ± 20
HMB	120 ± 23	64 ± 6	62 ± 8	107 ± 14	110 ± 12	51 ± 110
Main effect period						
Delivery	138 ± 9	64 ± 6	66 ± 17	118 ± 10	89 ± 20	54 ± 10
Lactation	109 ± 13	92 ± 30	58 ± 12	98 ± 11	106 ± 12	75 ± 23
treatment effect						
Control delivery (CONT-D)	138 ± 5	65 ± 6^{a}	76 ± 18^{b}	123 ± 5^{c}	73 ± 7	60 ± 7^{a}
Control lactation (CONT-L)	117 ± 5	120 ± 11^{b}	47 ± 3^{a}	96 ± 6^{a}	97 ± 5	95 ± 7^{b}
HMB delivery (HMB-D)	137 ± 12	63 ± 7^{a}	55 ± 3^{a}	113 ± 12^{bc}	105 ± 14	48 ± 10^{a}
HMB lactation (HMB-L)	102 ± 15	65 ± 5^{a}	69 ± 5^{b}	101 ± 14^{ab}	115 ± 8	54 ± 10^{a}
Pooled SEM	3.6	2.6	3.5	3.5	3.3	3.0
Main effects and interaction						
Supplementation (S)	0.042	<0.001	0.908	0.401	<0.001	<0.001
Period (P)	<0.001	<0.001	0.036	<0.001	<0.001	<0.001
S x P	0.069	<0.001	<0.001	0.047	0.050	<0.001

OPG: osteoprotegerin; OC: osteocalcin; BMP-2: bone morphogenetic protein 2; TIMP-2: tissue inhibitor of metalloproteinases 2; MMP8: matrix metalloproteinase 8;
MMP-13: matrix metalloproteinase 13.
CONT-D: control pregnant females at delivery; CONT-L: control pregnant females after the lactation period; HMB-D: pregnant HMB females at delivery; HMB-L pregnant HMB females after the lactation period.
Data shows the pixel value of 8-bit grey-scale digital images, with a scale from 0 (white pixel) to 255 (black pixel), where the higher the pixel value, the higher the intensity of the immunohistochemical reaction.
Data are presented as lsmeans ± standard deviation. SEM: standard error of the means. $^{a, b, c}$ - mean values in rows with different letters differ significantly at $P < 0.05$.

Table S2. Effect of HMB supplementation (0.02 g/kg b.w.) during the middle trimester of pregnancy (13–26 d) on the intensity of immunoreactions (pixel value) against bone turnover proteins in the trabecular bone of spiny mice femora.

Treatment	OPG	OC	BMP-2	TIMP-2	MMP-8	MMP-13
Main effect supplementation						
Control	119 ± 12	111 ± 16	88 ± 10	106 ± 9	80 ± 20	77 ± 13
HMB	113 ± 11	79 ± 10	74 ± 30	125 ± 14	66 ± 7	88 ± 25
Main effect period						
Delivery	119 ± 12	87 ± 13	62 ± 18	119 ± 19	61 ± 5	65 ± 3
Lactation	113 ± 11	102 ± 25	100 ± 6	112 ± 8	85 ± 15	101 ± 13
treatment effect						
Control delivery (CONT-D)	126 ± 12[b]	96 ± 2[b]	79 ± 4[b]	104 ± 9[a]	61 ± 6[a]	66 ± 4 [a]
Control lactation (CONT-L)	112 ± 5[a]	125 ± 7[c]	97 ± 3[c]	108 ± 8[a]	99 ± 2[c]	89 ± 7[b]
HMB delivery (HMB-D)	112 ± 7[a]	79 ± 13[a]	46 ± 5[a]	134 ± 13[b]	61 ± 3[a]	65 ± 3[a]
HMB lactation (HMB-L)	115 ± 15[ab]	79 ± 7[a]	103 ± 7[c]	115 ± 6[a]	72 ± 5[b]	112 ± 4[c]
Pooled SEM	3.7	2.9	1.7	3.4	1.6	1.7
Main effects and interaction						
Supplementation	0.121	<0.001	<0.001	<0.001	<0.001	<0.001
Period	0.133	<0.001	<0.001	0.043	<0.001	<0.001
S x P	0.026	<0.001	<0.001	0.002	<0.001	<0.001

OPG: osteoprotegerin; OC: osteocalcin; BMP-2: bone morphogenetic protein 2; TIMP-2: tissue inhibitor of metalloproteinases 2; MMP8: matrix metalloproteinase 8;
MMP-13: matrix metalloproteinase 13.
CONT-D: control pregnant females at delivery; CONT-L: control pregnant females after the lactation period; HMB-D: pregnant HMB females at delivery; HMB-L pregnant HMB females after the lactation period.
Data shows the pixel value of 8-bit grey-scale digital images, with a scale from 0 (white pixel) to 255 (black pixel), where the higher the pixel value, the higher the intensity of the immunohistochemical reaction.
Data are presented as lsmeans ± standard deviation. SEM: standard error of the means. [a, b, c] - mean values in rows with different letters differ significantly at P < 0.05.

Table S3. Effect of HMB supplementation (0.02 g/kg b.w.) during the middle trimester of pregnancy (13–26 d) on the intensity of immunoreactions (pixel value) against bone turnover proteins in the compact bone of spiny mice femora.

Treatment	OPG	OC	BMP-2	TIMP-2	MMP-8	MMP-13
Main effect supplementation						
Control	133 ± 16	119 ± 50	71 ± 11	131 ± 11	71 ± 19	88 ± 10
HMB	92 ± 13	78 ± 13	83 ± 14	123 ± 17	61 ± 8	90 ± 25
Main effect period						
Delivery	116 ± 19	80 ± 12	73 ± 11	118 ± 13	57 ± 7	80 ± 15
Lactation	108 ± 31	117 ± 52	81 ± 16	136 ± 10	75 ± 17	98 ± 19
treatment effect						
Control delivery (CONT-D)	130 ± 13^{c}	71 ± 7^{a}	74 ± 15^{a}	127 ± 10^{b}	59 ± 7	94 ± 8^{c}
Control lactation (CONT-L)	136 ± 19^{c}	168 ± 5^{c}	67 ± 4^{a}	135 ± 12^{b}	83 ± 21	81± 7^{b}
HMB delivery (HMB-D)	102 ± 10^{b}	89 ± 8^{b}	71 ± 7^{a}	109 ± 9^{a}	55 ± 6	66 ± 3^{a}
HMB lactation (HMB-L)	81 ± 5^{a}	67 ± 7^{a}	95 ± 11^{b}	137 ± 8^{b}	67 ± 4	114 ± 10^{d}
Pooled SEM	4.6	2.4	3.6	3.5	4.0	2.7
Main effects and interaction						
Supplementation	<0.001	<0.001	0.002	0.036	0.016	0.354
Period	0.098	<0.001	0.027	<0.001	<0.001	<0.001
S x P	0.008	<0.001	<0.001	0.007	0.174	<0.001

OPG: osteoprotegerin; OC: osteocalcin; BMP-2: bone morphogenetic protein 2; TIMP-2: tissue inhibitor of metalloproteinases 2; MMP8: matrix metalloproteinase 8;
MMP-13: matrix metalloproteinase 13.
CONT-D: control pregnant females at delivery; CONT-L: control pregnant females after the lactation period; HMB-D: pregnant HMB females at delivery; HMB-L pregnant HMB females after the lactation period.
Data shows the pixel value of 8-bit grey-scale digital images, with a scale from 0 (white pixel) to 255 (black pixel), where the higher the pixel value, the higher the intensity of the immunohistochemical reaction.
Data are presented as lsmeans ± standard deviation. SEM: standard error of the means. $^{a, b, c, d}$ - mean values in rows with different letters differ significantly at $P < 0.05$.

Table S4. Effect of HMB supplementation (0.02 g/kg b.w.) during the middle trimester of pregnancy (13–26 d) on the intensity of immunoreactions (pixel value) against bone turnover proteins in the articular cartilage of spiny mice femora.

Treatment	OPG	OC	BMP-2	TIMP-2	MMP-8	MMP-13
Main effect supplementation						
Control	193 ± 22	163 ± 38	124 ± 19	159 ± 13	86 ± 10	152 ± 31
HMB	208 ± 17	106 ± 14	148 ± 39	182 ± 16	109 ± 11	167 ± 19
Main effect period						
Delivery	208 ± 18	118 ± 14	125 ± 17	163 ± 14	94 ± 20	167 ± 19
Lactation	193 ± 21	152 ± 50	146 ± 41	177 ± 50	101 ± 9	152 ± 31
treatment effect						
Control delivery (CONT-D)	209 ± 16^{b}	127 ± 5^{b}	136 ± 18^{b}	153 ± 12	77 ± 6^{a}	173 ± 23^{c}
Control lactation (CONT-L)	179 ± 14^{a}	199 ± 9^{c}	112 ± 12^{a}	164 ± 11	94 ± 6^{b}	131 ± 21^{a}
HMB delivery (HMB-D)	206 ± 20^{b}	108 ± 12^{a}	115 ± 9^{a}	174 ± 7	111 ± 15^{c}	161 ± 13^{b}
HMB lactation (HMB-L)	210 ± 13^{b}	104 ± 15^{a}	180 ± 28^{c}	190 ± 19	108 ± 7^{c}	173 ± 23^{c}
Pooled SEM	5.6	3.9	6.8	4.6	3.2	7.3
Main effects and interaction						
Supplementation	0.014	<0.001	0.001	<0.001	<0.001	0.047
Period	0.018	<0.001	0.003	0.006	0.033	0.052
S x P	0.004	<0.001	<0.001	0.585	0.005	0.001

OPG: osteoprotegerin; OC: osteocalcin; BMP-2: bone morphogenetic protein 2; TIMP-2: tissue inhibitor of metalloproteinases 2; MMP8: matrix metalloproteinase 8;
MMP-13: matrix metalloproteinase 13.
CONT-D: control pregnant females at delivery; CONT-L: control pregnant females after the lactation period; HMB-D: pregnant HMB females at delivery; HMB-L pregnant HMB females after the lactation period.
Data shows the pixel value of 8-bit grey-scale digital images, with a scale from 0 (white pixel) to 255 (black pixel), where the higher the pixel value, the higher the intensity of the immunohistochemical reaction.
Data are presented as lsmeans ± standard deviation. SEM: standard error of the means. $^{a, b, c}$ - mean values in rows with different letters differ significantly at P < 0.05.
